# Comment on Chen et al. Dual Blockade of Lactate/GPR81 and PD-1/PD-L1 Pathways Enhances the Anti-Tumor Effects of Metformin. *Biomolecules* 2021, *11*, 1373

**DOI:** 10.3390/biom12040573

**Published:** 2022-04-13

**Authors:** Nicolai Stransky, Stephan M. Huber

**Affiliations:** Department of Radiation Oncology, University of Tübingen, Hoppe-Seyler-Str. 3, 72076 Tübingen, Germany; nicolai.stransky@med.uni-tuebingen.de

In the study of Chen et al. [1] the authors used 3-hydroxybutyric acid (3-OBA) as a specific GPR81 (hydroxycarboxylic acid receptor-1—HCA1) receptor antagonist. In their comment, Nezhady and Chemtob [2] comprehensively demonstrated that any direct evidence for a GPR81 receptor- (or signaling)-antagonizing action is lacking in the scientific literature. Instead, 3-OBA is an established ligand of the HCA2 receptor [3]. Since Chen et al. [1] built up their reasoning on experimental data solely obtained with the putative GPR81-inhibitor 3-OBA, part of their conclusions concerning the involvement of the GPR81 receptor or its downstream signaling may be in question. Nevertheless, we decided not to retract the study from publication for the following reasons.

Several studies (most of them quoted in the comment by Nezhady and Chemtob [2] to demonstrate the missing evidence for a GPR81 (HCA1) receptor-antagonizing action of 3-OBA) provide overwhelming evidence that 3-OBA antagonizes the cellular effects of extracellularly applied lactate or those of the established specific GPR81 agonist 3,5-dihydroxybenzoic acid (3,5-DHBA) [4] in various model systems. In particular, Khatib-Massalha et al. [5] have impressively demonstrated that 3-OBA mimics the effect of GPR81 knockout on lactate-induced upregulation of liver neutrophils and antagonizes lactate effects in vivo. Moreover, Shen et al. [6] have shown that 3-OBA blunts the hazardous effect of oxygen and glucose depletion on GPR81-transfected N2A cells, while lactate reverses this 3-OBA effect. Notably, a MEK inhibitor abolishes this protective 3-OBA action that might hint at an interference of 3-OBA with the MAP kinase pathway. As a matter of fact, lactate and the specific GPR81 agonist 3,5-DHBA reportedly increase myotube diameter in mouse C2C12 skeletal muscle cells in a MEK-inhibitor-sensitive manner [7]. In addition, 3-OBA has been proven to attenuate lactate-induced nuclear accumulation of β-arrestin2, p300 and CBP and to prevent lactate-induced phosphorylation of LATS1 and YAP, preserving YAP expression, to blunt lactate-promoted HMGB1 acetylation and to suppress lactate-increased exosomal HMGB1 levels in macrophages [8]. Furthermore, 3-OBA reportedly abolishes while the specific GPR81 agonist 3,5-DHBA mimics lactate effects in breast cancer cells [9], and finally, 3-OBA has been shown to abrogate lactate- and 3,5-DHBA-mediated processes in intestinal stem-cell-mediated epithelial development, mimicking effects of GPPR81 knockout [10].

Although proof of interference of 3-OBA with the HCA1 (GPR81) receptor directly or with its downstream signaling is missing, as legitimately criticized by Nezhady and Chemtob [2], this incomplete list of observations indicates that 3-OBA functionally blocks GPR81-triggered cellular effects. Most importantly, independently of the underlying mechanisms, the data in the study by Chen et al. [1] are clinically relevant, demonstrating the synergistic anti-tumor effect of 3-OBA, metformin and PD-1/PD-L1 blockade in vivo.

We want to thank Dr. Nezhady and Dr. Chemtob for bringing this issue to our attention.

## Data Availability

Not applicable.
